# Auf dem Weg zu einer nachhaltigen Mobilität

**DOI:** 10.1007/978-3-662-62195-0_7

**Published:** 2020-10-26

**Authors:** Carolin Zachäus, Benjamin Wilsch, Eyk Bösche, Martin Martens, Annette Randhahn

**Affiliations:** Institut für Innovation und Technik, Berlin, Germany; Institut für Innovation und Technik, VDI/VDE Innovation + Technik GmbH, 10623 Berlin, Deutschland

## Abstract

Mit der Entwicklung emissionsarmer alternativer Antriebstechnologien sowie einer zunehmenden Automatisierung und Digitalisierung der Fahrzeuge und Verkehrssysteme bestehen die technischen Möglichkeiten, eine nahtlose, nachhaltige Mobilität gleichzeitig sozial, ökologisch und ökonomisch gerecht umzusetzen. Jetzt gilt es, einzelne Mobilitätsangebote in einem effizienten Mobilitätssystem zusammenzufuhren, das nicht nur die vielfaltigen Anforderungen der Nutzer:innen möglichst gut bedienen kann, sondern vor allem ein Erreichen der Nachhaltigkeitsziele ermöglicht und vorantreibt.

Der Verkehr trägt zu einem Viertel an den $CO_2$-Emissionen in Europa bei. Insbesondere der Straßenverkehr ist zudem Ursache für hohe lokale Luftverschmutzungen in Städten mit direkten Auswirkungen auf die Gesundheit und Lebensqualität der meisten EU-Bürger:innen. Der EU Green Deal der Europäischen Kommission sieht eine Einsparung von 90 Prozent der verkehrsbedingten $CO_2$-Emissionen bis 2050 vor. In Deutschland liegt das Ziel für 2030 bei 98 Millionen Tonnen $CO_2$-Äquivalenten pro Jahr (Bundesministerium für Umwelt, Naturschutz und nukleare Sicherheit 2018). Der Anstieg der Emissionen im Verkehrsbereich nach 1990 ist vor allem Resultat der voranschreitenden Globalisierung, des Bevölkerungswachstums, aber auch der Urbanisierung, die in vielen Ländern von einem erweiterten Zugang zu verschiedenen Mobilitätsformen begleitet wurde. Damit einher ging eine Steigerung der Mobilität jedes Einzelnen sowie des Güterverkehrs (Intraplan Consult GmbH 2014; Nobis et al. 2019).

Zwar konnte im Zuge der Verkehrsentwicklung seit Mitte des 20. Jahrhunderts weltweit die individuelle Mobilität verbessert werden, allerdings wurden die Auswirkungen auf Gesellschaft und Klima nicht ausreichend berücksichtigt. Eine allgemein gestiegene Mobilitätsnachfrage führte zusammen mit einer anhaltend dominierenden Autonutzung[40] unvermeidbar zu einer Überbeanspruchung des Verkehrsraums und damit zu stetig steigenden Umweltbelastungen. Neben $CO_2$-Emissionen müssen allerdings auch weitere Kriterien wie Lärm- und Schadstoffbelastung (Stickoxide: $NO_x$, Feinstaub), Verkehrsunfälle und -tote, Infrastruktur- und Betriebskosten, Reisezeit,

*[40] 58 Prozent der in 2017 in Deutschland zurückgelegten Wege war motorisierter Individualverkehr (Nobis und Kuhnimhof 2018).*

Vernichtung von Lebens- und Erholungsräumen durch erhöhte Raumnutzung, Inklusion sowie der Gesundheitsnutzen in die Betrachtung verschiedener Mobilitätsformen einfließen (Umweltbundesamt 2019a, 2019b; Statistisches Bundesamt 2019). Beispielsweise ergeben sich im gesamtwirtschaftlichen Vergleich für den Radverkehr geringere Gesamtkosten und ein externer Nutzen, während der Pkw-Verkehr externe Kosten verursacht[41]. Nicht nur der Personenverkehr, sondern auch der Güterverkehr trägt zu einem großen Teil der $NO_X$-Emissionen (zum Beispiel 31 Prozent in Berlin) und zu 20 Prozent der tödlichen Verkehrsunfälle 2017 in Deutschland bei (Agora Verkehrswende 2019a). Insgesamt werden in Deutschland durch den Straßenverkehr Umweltkosten in Höhe von 52 Milliarden Euro im Jahr verursacht (Umweltbundesamt 2016).

Die Anforderungen an die Mobilität lassen sich anhand vieler Kriterien differenzieren – beispielsweise nach Land und Entwicklungsstand, nach Alter oder Einkommen – und können sehr unterschiedlich ausfallen. Das Zusammenleben vieler Menschen in Städten sorgt für eine besonders hohe gesundheitliche Belastung durch Schadstoffe (Krzyzanowski und Kuna-Dibbert 2005; Wu et al. 2020), und Lärm sowie der Platzbedarf für Mobilitätsformen wirken sich direkt auf die Lebensqualität in urbanen Räumen aus. Da diese Lebensqualität aber auch eng mit der individuellen Mobilität verknüpft ist, muss eine nachhaltige Mobilität möglichst gesundheits- und klimaschonend sowie inklusiv sein. Die deutlichen Unterschiede in den Gesamtnutzungskosten der unterschiedlichen Mobilitätsformen führt bereits dazu, dass das Angebot für jeden Einzelnen zunächst von seinem Wohlstand abhängt.

Um eine gleichzeitig sozial und ökologisch gerechte sowie ökonomisch tragfähige Umsetzung einer nahtlosen, nachhaltigen Mobilität zu erreichen, sind sowohl eine grundsätzliche Verkehrs- bzw. Mobilitätswende als auch die Ableitung spezifischer Lösungen für einzelne Gebiete und Regionen erforderlich. Eine erste Differenzierung kann für den urbanen und den ländlichen Raum erfolgen. Menschen in Städten leiden besonders unter den bereits angeführten Negativauswirkungen des Verkehrs. Im urbanen Verkehr werden allerdings schon Maßnahmen zur Stärkung der Elektromobilität sowie zur Nutzung alternativer Kraftstoffe, des öffentlichen Personennahverkehrs (ÖPNV) und des Fuß- und Radverkehrs ergriffen. Auch werden sie durch die Einführung und Umsetzung intelligenter digitaler Verkehrssysteme und zukünftig das automatisierte Fahren unterstützt. Für den ländlichen Raum und die besonderen Anforderungen der Menschen dort müssen aber ebenfalls geeignete Lösungen

[41] *Gesamtkosten 1,55 ct/Fahrrad-km und 98,38 ct/Pkw-km; externer Nutzen von 81,47 ct/Fahrrad-km gegenüber externen Kosten von 4,35 ct/Pkw-km (Trunk 2010). Externe Kosten von 11 ct/Pkw-km und externer Nutzen von 18 ct/Fahrrad-km bzw. 37 ct/Fußweg-km (Gössling et al. 2019).*

gefunden werden – nicht zuletzt, um die möglichen positiven Auswirkungen der Digitalisierung und (Fahrzeug-)Automatisierung auf die Attraktivität dieser Gebiete vollständig zu erschließen. Erschwert wird der Einsatz digitaler Technologien im ländlichen Raum derzeit noch durch eine mangelhafte Netzabdeckung. Außerdem ist das Mobilitätsangebot auf dem Land insgesamt noch sehr begrenzt.

Im Folgenden werden die Potenziale emissionsarmer Antriebe sowie von Digitalisierungs- und Automatisierungsmaßnahmen zur Umsetzung einer nachhaltigen Mobilität am Beispiel Deutschland erläutert und bezüglich ihres Beitrages zum Klima- und Umweltschutz und zur Bewältigung offener Herausforderungen diskutiert sowie konkrete Umsetzungsbeispiele vorgestellt. Anschließend werden die Themenkreise im Hinblick auf Synergien miteinander und innerhalb des gesamten Verkehrssystems betrachtet. Es wird gezeigt, welche Voraussetzungen für die dauerhaft erfolgreiche Umsetzung einer nachhaltigen und gleichzeitig nutzungsfreundlichen Mobilität geschaffen werden müssen.

## *Elektrifizierung und alternative Kraftstoffe*

Der Fahrzeugbestand in der Europäischen Union umfasste 2018 rund 268 Millionen Personenkraftwagen sowie 40 Millionen Nutzfahrzeuge, bei denen der Anteil alternativer Antriebe seit 2007 stetig steigt. Dieser Trend wird in den kommenden Jahren deutlich zunehmen, da die EU entsprechende Maßnahmen zur Reduzierung der $CO_2$-Emissionen ergriffen hat.

Die Neuregelung der EU für Pkw und leichte Nutzfahrzeuge soll dafür sorgen, dass Neuwagen ab 2030 durchschnittlich 37,5 Prozent weniger $CO_2$ im Vergleich zu 2021 ausstoßen (Europäische Kommission 2019a). Bei schweren Nutzfahrzeugen ist eine Reduktion der $CO_2$-Emissionen von neuzugelassenen Fahrzeugen bis 2030 um 30 Prozent gegenüber dem Stand von 2019 vorgesehen (Europäische Kommission 2019b).

Um die gesetzten Ziele zur $CO_2$-Minderung bei Pkw und leichten Nutzfahrzeugen zu erreichen, bedeutet dies gemäß einer Studie des McKinsey Center for Future Mobility, dass die jährlichen Neuzulassungen elektrisch aufladbarer Fahrzeuge (batterieelektrische Autos und Plug-in-Hybride) von rund 0,33 Millionen im Jahr 2018[42], bzw. 0,49 Millionen im Jahr 2019[43], auf bis zu 6,2 Millionen im Jahr 2030 ansteigen. Anders gesagt: Der Gesamtbestand von rund einer Million Fahrzeuge im Jahr 2018 müsste auf etwa 33 Millionen Fahrzeuge anwachsen (Cornet et al. 2019).

[42] *Davon circa 0,31 Millionen Pkw und circa 0,02 Millionen leichte Nutzfahrzeuge (ACEA).*

[43] *Davon circa 0,46 Millionen Pkw und circa 0,03 Millionen leichte Nutzfahrzeuge (ACEA).*

Die $CO_2$-Emissionen schwerer Nutzfahrzeuge und Busse machen in der EU rund 6 Prozent aller $CO_2$-Emissionen und rund 27 Prozent der $CO_2$-Emissionen des Straßenverkehrs aus (Rat der Europäischen Union 2019). Bei den schweren Nutzfahrzeugen lag der Anteil der mit Diesel betriebenen zugelassenen mittelschweren und schweren Lastkraftwagen (mehr als 3,5 Tonnen) in der EU bei 97,9 Prozent, während der Anteil benzinbetriebener bei nur 0,1 Prozent lag. Der Anteil alternativer Antriebe (APV) lag insgesamt bei 2 Prozent des EU-Marktes. Dabei machten elektrisch aufladbare Fahrzeuge (ECV[44]) 0,2 Prozent der gesamten Neuzulassungen aus. Die Neuzulassungen von ECV stiegen somit von 357 Lkw im Jahr 2018 auf 747 im Jahr 2019. Der überwiegende Anteil schwerer Nutzfahrzeuge mit alternativen Kraftstoffen entfällt auf Erdgas betriebene Fahrzeuge[45] (European Automobile Manufacturers' Association 2020b).

Im Bereich der Busse lag der Anteil der mit Diesel betriebenen zugelassenen mittelschweren und schweren Busse (über 3,5 Tonnen) in der EU bei 85 Prozent, während der Anteil benzinbetriebener bei nahe Null lag. APVs hatten insgesamt einen Anteil von 15 Prozent des EU-Marktes, wobei ECVs 4 Prozent der gesamten Neuzulassungen ausmachten. Die Neuzulassungen von ECVs stiegen somit von 594 Bussen im Jahr 2018 auf 1607 im Jahr 2019. Hybridbusse (HEV) hatten einen Marktanteil von 4,8 Prozent. Der verbleibende Anteil von 6,2 Prozent entfällt überwiegend auf erdgasbetriebene Busse (European Automobile Manufacturers' Association 2020a).

Dies bedeutet, dass im Verkehrssektor noch immer zu mehr als 90 Prozent Kraftstoffe aus Mineralöl verwendet werden. Biokraftstoffe und Strom spielen bislang nur eine untergeordnete Rolle.

*Nachhaltigkeit alternativer Antriebstechnologien und Kraftstoffe*

Es gibt inzwischen zahlreiche Werkzeuge, um $CO_2$-Emissionen im Straßenverkehr zu reduzieren; sie ermöglichen den Übergang zu emissionsarmen alternativen Antrieben ebenso wie eine weitere Steigerung der Effizienz konventioneller Fahrzeuge. In allen Fällen treibt Strom aus erneuerbaren Quellen die Autos entweder direkt oder indirekt an.

Mehrere Optionen zur direkten und indirekten Nutzung von Strom im Verkehr sind denkbar (Blanck et al. 2013):

- direkte Nutzung von Strom ohne Zwischenspeicherung (zum Beispiel über Oberleitungen) oder mit Zwischenspeicherung (zum Beispiel in Batterien von elektrisch betriebenen Fahrzeugen)

[44] *Elektrisch aufladbare Fahrzeuge (ECV) umfassen batterielektrische Elektrofahrzeuge, Brennstoffzellen-Elektrofahrzeuge, Fahrzeuge mit Range Extender und Plug-in-Hybride.*

[45] *98 Prozent davon waren mit Erdgas betriebene Fahrzeuge.*

- indirekte Nutzung von Strom zur Erzeugung von Kraftstoffen [zum Beispiel flüssige Kraftstoffe (Power-to-Liquid: PtL), gasförmige Kraftstoffe (Power-to-Gas: PtG) und Wasserstoff (Power-to-Hydrogen: $PtH_2$)].

Beide Entwicklungswege unterscheiden sich teilweise sehr hinsichtlich Nutzungsrestriktionen, Energieeffizienz, Entwicklungsstand der Anwendungstechnologien, benötigter Infrastruktur, Möglichkeiten zur Stromspeicherung und Kosten. Dies ist insofern von Bedeutung, dass der Verkehr nicht der einzige Sektor ist, der zur Erreichung der Klimaschutzziele von fossilen Brennstoffen, entweder flüssig oder gasförmig, auf erneuerbar erzeugten Strom umsteigen muss, entweder als Energieträger oder als Rohstoff zur Herstellung von Brennstoffen. Wegen des hohen spezifischen Stromverbrauchs für die Herstellung strombasierter Energieträger ist die Art und Weise der Stromerzeugung der entscheidende Faktor für die Treibhausgasemission. Der weitere Ausbau erneuerbarer Energiequellen verbessert nicht nur die Klimabilanz neuzugelassener batterieelektrischer Fahrzeuge, sondern verringert auch die Treibhausgasemissionen bei der Herstellung von PtL, PtG und $PtH_2$.

Letztendlich gilt der Grundsatz „Efficiency First". Technisch gesehen ist eine direkte Nutzung erneuerbarer Energiequellen oft effizienter und kostengünstiger als die indirekte Nutzung von Strom zur Erzeugung von Kraftstoffen wie PtL, PtG oder $PtH_2$. Bei der Stromumwandlung ist besonders auf die Stromherkunft zu achten. Der Unterschied zwischen den Optionen zur direkten und indirekten Nutzung von Strom im Verkehr kann beim Strombedarf mehrere Größenordnungen betragen (Transport & Environment 2018). Abb. 7.1 zeigt die Effizienz der Nutzung von erneuerbarem Strom im Pkw unter Berücksichtigung möglicher zukünftiger Effizienzgewinne.

Die Effizienz der Nutzung von Strom aus erneuerbaren Energiequellen spiegelt sich direkt in den Treibhausgasemissionen der verschiedenen Antriebstechnologien wider. So zeigt die Studie „Klimabilanz von strombasierten Antrieben und Kraftstoffen" (Agora Verkehrswende 2019b), dass ein Fahrzeug der Kompaktklasse mit Brennstoffzelle und elektrolytisch hergestelltem Wasserstoff aus deutschem Strommix (indirekte Nutzung von Strom) nach einer Fahrleistung von 150.000 Kilometern 75 Prozent mehr Treibhausgasemissionen verursacht als ein nur mit Batterie betriebener Pkw mit 35 kWh Batteriekapazität (direkte Nutzung von Strom mit Zwischenspeicherung). Im Vergleich zu einem Dieselfahrzeug liegen die Treibhausgasemissionen eines mit Wasserstoff betriebenen Fahrzeugs um rund 50 Prozent höher.

Ganz allgemein lässt sich der Vorteil von „Efficiency First", hier also einer direkten Stromnutzung gegenüber einer indirekten Stromnutzung, auch auf die weiteren Verkehrsträger auf der Straße übertragen. Die Realisierung von Energieeffizienzpotenzialen ist somit essenziell für eine Reduktion der Treibhausgase im Verkehr. Weitreichende Maßnahmen, basierend auf direkter oder indirekter Elektrifizierung, ermöglichen eine nachhaltige Mobilität (Wietschel et al. 2018). Im Personen- und

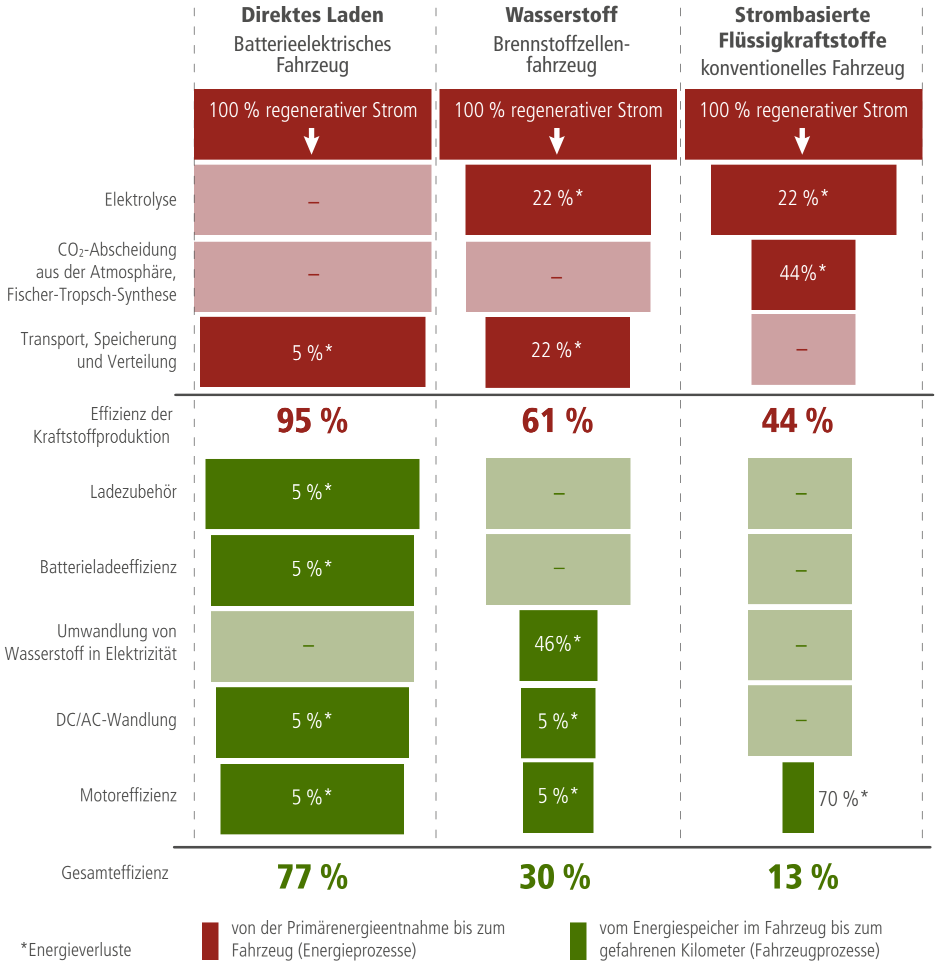

*Abb. 7.1 Effizienz verschiedener Pkw-Technologiepfade basierend auf erneuerbar erzeugtem Strom. (Eigene Darstellung nach Transport & Environment 2018)*

leichten Güterverkehr spielen vor allem batterieelektrische Fahrzeuge eine große Rolle (Ziel 2030: 10 bis 30 Prozent Pkw im Bestand, 2050: 30 bis 70 Prozent). Zudem kann der Schienenverkehr für bestimmte Technologien wie die Brennstoffzelle ein wichtiger Einstiegsmarkt sein. Im Bereich schwerer Nutzfahrzeuge bieten batterieelektrische Antriebe (Nahverkehr) und Oberleitung, Wasserstoff und synthetische Kraftstoffe (Personenfern- und Güterverkehr) die besten Einsatzmöglichkeiten (Wietschel et al. 2018).

*Batterieelektrische Busse im ÖPNV*

Auch wenn der straßengebundene ÖPNV an den gesamten Verkehrsemissionen nur einen geringen Anteil hat, so ist dieser in absoluten Zahlen nicht zu vernachlässigen. Zudem nimmt der Nahverkehrsbus mit 80 Gramm $CO_2$-Ausstoß pro Personenkilometer Platz drei im Vergleich aller Personenbeförderungsmittel nach Flugzeug und Pkw ein (Umweltbundesamt 2020). Darüber hinaus bestehen ÖPNV-Busflotten in Deutschland und Europa nicht unbedingt aus den neuesten modernen „EURO VI"-Dieselfahrzeugen. Bei einer üblichen Fahrzeugnutzungsdauer zwischen zehn und 15 Jahren sind auch heute noch ohne Weiteres „EURO II–V"-Fahrzeuge in den Nahverkehrsflotten zu finden, die nicht unwesentlich zur Belastung der Innenstädte mit $NO_X$ und Feinstaub beitragen.

Deshalb lohnt sich ein genauerer Blick auf die Klima- und Umweltentlastungspotenziale von rein batterieelektrischen Bussen im ÖPNV. Unter der Annahme, dass ein moderner 12-Meter-Dieselbus im städtischen Nahverkehr rund 60.000 Kilometer zurücklegt und 40 Liter Treibstoff pro Kilometer verbraucht, kann dessen Ersatz mit einem rein batterieelektrisch betriebenen Bus etwa 61 Tonnen $CO_2$ pro Jahr einsparen.[46] Die gesamte Nahverkehrsbusflotte in Deutschland besteht aus rund 45.600 Fahrzeugen (Statistisches Bundesamt 2020). Würde nur die Hälfte elektrisch fahren, würde der $CO_2$-Ausstoß aus dem straßengebundenen ÖPNV um mehr als 1,4 Millionen Tonnen jährlich sinken. Dies gilt natürlich wie bei anderen Fahrzeugen auch nur dann, wenn der Strom für die Elektrobusse zu 100 Prozent aus erneuerbaren Energiequellen stammt.

Der Betrieb der Elektrobusse ist darüber hinaus lokal schadstoffemissionsfrei, vorausgesetzt es gibt keine mit fossilen Kraftstoffen betriebenen Nebenaggregate. Ein weiterer wesentlicher Vorteil ist der geräuschlose Elektromotor, der insbesondere bei der Anfahrt der Busse für eine bedeutende Lärmreduzierung sorgt. Insgesamt können Elektrobusse also vor allem in Ballungsräumen nicht nur zum Klimaschutz, sondern wesentlich zur Verbesserung der Lebensqualität beitragen.

*Oberleitungsgebundene schwere Nutzfahrzeuge*

Im Jahr 2018 hatte der Güterverkehr auf der Straße in Deutschland einen Anteil von über 70 Prozent an der Gesamttransportleistung (Allianz pro Schiene 2020). Zwar ist ein deutlicher Ausbau des Schienengüterverkehrs sowohl durch Erweiterung der Infrastruktur als auch durch Verdichtung des Verkehrs zu erwarten, doch dieser Effekt wird nach Prognosen des Umweltbundesamtes (Bergk et al. 2016) durch eine deutliche Zunahme des Gesamtgüterverkehrs weitestgehend kompensiert. Demnach

[46] *Umrechnungsfaktor 2,56 kg/Liter Diesel.*

werden selbst im optimalen Szenario im Jahr 2050 weiterhin mindestens 60 Prozent der Güter auf der Straße transportiert. Dies zeigt, dass auch im straßengebundenen Güterverkehr ein Wechsel von den bisher dominanten dieselbetriebenen Fahrzeugen hin zu alternativen Antrieben notwendig ist, um die Ziele der Bundesregierung zur Verringerung der $CO_2$-Emissionen von 42 Prozent im Vergleich zu 1990 bis 2030 erreichen zu können.

Wie bereits dargelegt, ist die direkte Nutzung von Strom aus erneuerbaren Energien deutlich effizienter als die indirekte Nutzung, wie bei PtL, PtG oder $PtH_2$. Begrenzte Reichweiten, lange Ladezeiten und hohes Gewicht von Batterien machen jedoch die Nutzung von batterieelektrischen Fahrzeugen in vielen Anwendungsszenarien für Speditionen unwirtschaftlich. Eine direkte Bereitstellung von Strom über Oberleitungen an den Fahrbahnen ermöglicht hingegen die effektive Nutzung von klimaneutralem Strom aus erneuerbaren Energien. Dafür werden im Fahrzeug auch nur vergleichsweise kleine Batterien benötigt.

Derzeit wird die Nutzung von Oberleitungen für den Güterverkehr auf zwei jeweils fünf Kilometer langen Teststrecken auf der A5 in Hessen (Hessen Mobil – Straßen- und Verkehrsmanagement 2020) sowie der A1 in Schleswig-Holstein (Forschungs- und Entwicklungszentrum Fachhochschule Kiel GmbH 2020) unter Realbedingungen erprobt. Eine weitere Teststrecke wird auf der B462 in Baden-Württemberg (Ministerium für Verkehr Baden-Württemberg 2020) voraussichtlich im Jahr 2020 fertiggestellt. Im Rahmen der vom Bundesministerium für Umwelt, Naturschutz und nukleare Sicherheit geförderten Forschungsprojekte nutzen Speditionen Oberleitungs-Hybrid-Lkw mit Dieselmotor. Die Fahrzeuge können mit einem Pantografen an die Oberleitung ankoppeln und elektrisch fahren sowie die Batterien laden. Nicht mit Oberleitungen ausgestattete Streckenabschnitte können entweder mit geladener Batterie oder mit dem Dieselantrieb überbrückt werden.

Denkbar sind je nach Anwendungsfall auch Hybridvarianten mit alternativen Antrieben wie Brennstoffzellen oder rein batterieelektrische Kombinationen aus Pantograf und Batterie mit größerer Kapazität sowie die Nutzung der Oberleitung durch weitere Fahrzeugklassen wie etwa Fernbusse. Erste Ergebnisse der Feldtests zeigen die Praxistauglichkeit des Systems. Entsprechend einer Studie des Öko-Instituts (Hacker et al. 2020) könnten durch die Elektrifizierung eines etwa 4000 Kilometer langen Kernnetzes der insgesamt 13.000 Kilometer Autobahn in Deutschland eine jährliche $CO_2$-Ersparnis von bis zu 6 Millionen Tonnen bis 2030 erreicht werden. Bis zum Jahr 2040 wird ein Minderungspotenzial von bis zu 12 Millionen Tonnen jährlich genannt, was mehr als ein Drittel der aktuellen Emissionen des schweren Straßengüterverkehrs wäre.

## *Digitalisierung und Automatisierung*

Neben der Anwendung emissionsarmer und effizienter Antriebstechnologien lassen sich mit Hilfe von Digitalisierung und Automatisierung umfangreiche Potenziale einer nachhaltigeren Mobilität erschließen. Entsprechende Maßnahmen können sowohl die Wirkung emissionsarmer Antriebe verbessern – beispielsweise durch die Einführung und Optimierung der Elektrobuseinsatzplanung im Rahmen eines digitalen Betriebsmanagements – als auch die Nutzung umweltfreundlicher und geteilter Mobilitätsformen steigern.

Digitalisierung hat sich vor allem im Laufe der 2000er-Jahre als zentrales Modernisierungswerkzeug etabliert und die Wirksamkeit ist mit der Verfügbarkeit neuer Technologien stetig gestiegen – im Mittelpunkt steht dabei das mittlerweile omnipräsente Smartphone. Damit einher gehen scheinbar unendlichen Datenmengen, die wiederum durch entsprechende Hard- und Softwareentwicklungen, bis hin zu Methoden der Künstlichen Intelligenz (KI), zahlreiche neue Anwendungen ermöglicht haben.

Digitalisierung ist aber nicht zwangsläufig nachhaltig – welche Möglichkeiten Digitalisierungsmaßnahmen im Verkehrsbereich bieten und welche Besonderheiten bei der nachhaltigen Gestaltung beachtet werden müssen, kann anhand der folgenden Beispiele verdeutlicht werden:

*Verkehrsmanagement*

Dank einer weitreichenden Automatisierung der Verkehrsmengenerfassung verfügen insbesondere Städte zeitlich und örtlich über eine präzise Kenntnis der Verkehrslage. Zusammen mit der zunehmenden Vernetzung von Verkehrsinfrastruktur (vor allem Lichtsignalanlagen) und der Modernisierung von Verkehrsleitzentralen kann die Verkehrssteuerung (quasi-)instantan und anhand komplexer Verkehrsmodelle erfolgen. Die somit ermöglichte Verflüssigung des Verkehrs kann, ohne weitere Steuerungsparameter und -vorgaben, die Attraktivität des motorisierten Individualverkehrs steigern. Unter Berücksichtigung weiterer Umweltparameter (Meteorologie- und Luftqualitätsdaten) oder mittels der Bevorrechtigung des ÖPNV wird in vielen Städten jedoch bereits heute eine umweltsensitive Verkehrssteuerung umgesetzt, um sicherzustellen, dass die Verkehrsbelastung vor allem in begrenzten urbanen Räumen auf ein Minimum reduziert wird. Mit dem weiteren Ausbau einer Fahrzeug-zu-Fahrzeug- und Fahrzeug-zu-Infrastruktur-Vernetzung und der entsprechenden Fahrzeugautomatisierung lässt sich künftig eine noch höhere Koordination und Effizienz im Straßenverkehr erreichen.

*Digitalisierung des ÖPNV*

Als umweltfreundlichere und platzsparende Alternative zum motorisierten Individualverkehr (MIV) bietet der ÖPNV in Kommunen ein großes Potenzial als nachhaltige Mobilitätslösung. Viele Digitalisierungsmaßnahmen zielen auf die Steigerung der Attraktivität des ÖPNV ab, um Anreize für den Umstieg vom motorisierten Individualverkehr (MIV) zu setzen. Breite Anwendung finden inzwischen zum Beispiel „On-Demand"-Verkehre. Ein wesentlicher Nachteil des ÖPNV im Vergleich zum MIV beruht auf der mangelnden Abdeckung von „Door-to-door"-Reiserouten. Diese Lücke kann geschlossen werden, indem einzelne Wohnadressen oder ganze Bediengebiete mit Ruffahrzeugen angeschlossen werden. Solche mit dem Smartphone buchbaren Angebote werden bereits in vielen deutschen Städten erprobt und entweder als Zusatzangebot im Tarifverbund oder für eine geringe Nutzungspauschale bereitgestellt. Derartige Angebote können sowohl innerstädtischen Verkehr als auch Pendlerverkehr aus Randgebieten entlasten und zudem die Mobilität in ländlichen Regionen fördern. Nicht zuletzt für Bevölkerungsgruppen mit eingeschränkter Mobilität, wie Ältere oder Kranke, kann dies die Lebensqualität erheblich erhöhen. Der ökologische Mehrwert ist besonders groß, wenn Fahranfragen kombiniert werden und es somit zu einem „Ride Sharing" statt zum „Ride Hailing" kommt. On-Demand-Angebote können zudem zukünftig durch den Einsatz automatisierter Fahrzeuge deutlich erweitert werden, erprobt werden solche Angebote bereits an vielen Standorten (Verband Deutscher Verkehrsunternehmen 2020).

*Fahrzeugautomatisierung*

Im Zuge der Digitalisierung lässt sich auch die Automatisierung des Straßenverkehrs weiter vorantreiben (European Commission 2019). Die entsprechenden Themen in Forschung und Entwicklung reichen von der Sensorik und Verarbeitung der Datenflut mit Methoden der KI über Planung und Steuerung, Vernetzung, Sicherheit und Validierung bis hin zu rechtlichen, wirtschaftlichen, sozialen und ethischen Fragestellungen und schließlich zu grundsätzlichen Überlegungen zur Mensch-Maschine-Interaktion (COSMOS 2020). Zunächst leistet die Umstellung von konventionellen Fahrzeugen zu automatisierten Fahrzeugen – bis auf eventuelle Effizienzsteigerungen durch ein besseres Verkehrsmanagement – keinen signifikanten Beitrag zur Umweltverbesserung. Autonome Fahrzeuge, die immer und überall verfügbar sind, können sogar eine deutliche Zunahme des Straßenverkehrs und somit eine hinsichtlich der Nachhaltigkeitsziele konträre Entwicklung bewirken (Kellett et al. 2019). Allerdings birgt Automatisierung das Potenzial, die Gesamtzahl der Fahrzeuge signifikant zu verringern: Mit nur 3 Prozent der heute vorhandenen Fahrzeugflotte ließen sich die Mobilitätsbedürfnisse abdecken und somit Flächen- und Energieverbrauch sowie die Emissionen im Verkehr deutlich reduzieren. Dies setzt freilich voraus, die Fahrzeuge zu teilen und gemeinsam zu nutzen (Agora Verkehrswende 2017). Die Fahrzeugautomatisierung kann zudem dazu beitragen, Mobilität inklusiver zu gestalten, die Sicherheit im Straßenverkehr zu erhöhen und in der Folge die ökonomischen Kosten deutlich zu reduzieren sowie den Platzbedarf für den Verkehr effizienter zu gestalten (Wittpahl 2019).

Die angeführten Beispiele zeigen, dass Automatisierungs- und Digitalisierungsmaßnahmen für die Steigerung der Umweltverträglichkeit und des gesellschaftlichen Nutzens der Mobilität große Optimierungspotenziale bieten, zum Teil aber auch nicht nachhaltige Entwicklungen bewirken können. Für die Erreichung der Nachhaltigkeitsziele im Einklang mit der Erfüllung der Bedürfnisse der Nutzer:innen sowie zur Ableitung von Handlungsbedarfen und -empfehlungen ist daher eine Systembetrachtung erforderlich.

## *Multimodalität für ein Gesamtsystem Mobilität*

Im vergangenen Jahrzehnt kam es auf Grundlage bezahlbarer Digitaltechnik geradezu zu einer Explosion von neuartigen Mobilitätsangeboten. Dazu zählen insbesondere Ride-, Car-, Bike- und Scooter-Sharing sowie elektrische Tretroller. In der Folge entstand ein multimodales Angebot, das den Kunden deutlich mehr Optionen als bislang für den Weg von A nach B bietet. Um nun unterschiedliche Verkehrsmittel zu kombinieren, werden zentrale Plattformen und spezielle Apps entwickelt, mit denen sich zukünftig dann anbieterübergreifend multimodale Routen planen, buchen und abrechnen lassen. Solche Plattformen eignen sich hervorragend dazu, umweltfreundliche Reiserouten durch geringe Nutzungskosten zu priorisieren – wenn es gelingt, die Preise der unterschiedlichen Mobilitätsformen an ihre gesamtwirtschaftlichen Kosten zu binden. In vielen Städten wird die Nutzung multimodaler Angebote schon durch die Bereitstellung von Umsteigepunkten unterstützt. Beispielsweise ermöglichen Mobilitäts-Hubs in Hamburg („switchh“) und Berlin („Jelbi-Stationen“) an Stationen des ÖPNV-Netzes den Umstieg zwischen ÖPNV und Sharing-Angeboten.

Laut der Studie Mobilität in Deutschland (Nobis 2019) benutzten im Jahr 2017 nur 36 Prozent der befragten Personengruppen mehr als ein Verkehrsmittel. Im Gegensatz dazu zeigt sich mit 45 Prozent auch hier eine klare Präferenz zur individuellen Autonutzung. Um diesem Trend entgegenzuwirken und ein nachhaltiges Mobilitätsverhalten zu fördern, ist eine Verschiebung weg vom Individualverkehr und somit hin zu einer Gesamtsystemlösung auf Basis von multimodalen Plattformen anstrebenswert. Dies eröffnet die Chance einer komfortablen, nahtlosen, Nutzer:innen-orientierten Nutzung verschiedener Mobilitätsformen (u. a. öffentliche Verkehrsmittel, Sharing-Dienste, aktive Verkehrsmodi) innerhalb eines Weges.

Besonders aktive Verkehrsmodi wie Gehen und Radfahren sind entscheidend für die Entwicklung einer nachhaltigen Mobilität. Tatsächlich gewinnen Pedelec und Lastenrad zunehmend an Interesse und erweitern sowohl den Nutzungsradius als auch den Nutzungszweck von konventionellen Fahrrädern (Nobis 2019). In einer „Stadt der kurzen Wege" mit Zonen statt linearer Infrastruktur, mit verbesserter Nahraumversorgung und stärkerer Nutzungsdurchmischung werden notwendige Verkehrsleistungen reduziert und aktive Verkehrsmodi besonders gefördert (Brunsing 1999). Neben Investitionen in alternative Verkehrsmittel durch ein verbessertes Angebot des öffentlichen Nahverkehrs, sichere und flächendeckende Radverkehrsnetze sowie Abstellinfrastruktur, Förderung von Sharing-Angeboten, die Vernetzung der Verkehrsmittel und die Schaffung attraktiver Fußwege (vgl. Agora Verkehrswende 2017:26 ff) muss die Politik eine an den Nachhaltigkeitszielen orientierte Mobilitätsnutzung fördern. Die Verschärfung von Umweltzonen und Fahrverboten, die Reduzierung von Parkplätzen und die Bepreisung von klimaschädlichem Verkehr sowie die Einführung von Tempolimits können bei einem Einsparungspotenzial von bis zu 25 Millionen Tonnen Treibhausgasen im Jahr 2030 die Verlagerung weg vom privaten Fahrzeug hin zur Multimodalität begünstigen (Agora Verkehrswende 2018, 2017).

Im Güterverkehr können urbane Umschlagflächen, gemeinsame Kurierplattformen sowie gemeinsamer Personen- und Warentransport, intermodale urbane Logistik, urbanes Parkraummanagement und innovative Lösungen für die letzte Meile[47] zu einer signifikanten Steigerung der Nachhaltigkeit führen (Mobility4EU 2017; Agora Verkehrswende 2019b). Um Multimodalität als wichtigen Schritt zur nachhaltigen Mobilität zu fördern, sollten aktive Modi in der Verkehrsführung mit Vorrang umgesetzt werden, gefolgt von öffentlichen Verkehrsmitteln, Lieferverkehr und an letzter Stelle dem privaten Fahrzeug (National Association of City Transportation Officials 2019). Die Priorisierung kann durch intelligente und vernetzte Verkehrssteuerung unterstützt und tageszeitenabhängig angepasst werden.

## *Umsetzungsperspektiven und Fazit*

Das Gesamtsystem Mobilität – bestehend aus Elektrifizierung, Multimodalität (inkl. Sharing-Angeboten), Automatisierung und Digitalisierung (siehe Abb. 7.2) – kann nur im Zusammenspiel der Maßnahmen, durch Ausrichtung an gesellschaftlichen Zielen sowie unter Nutzung moderner Technologien effizient und nachhaltig betrieben werden. Notwendige und überwiegend bereits verfügbare Technologien sind digitale Plattformen zur Integration und zur Abwicklung von Buchungs- und Zahlungsvorgängen, intelligentes Verkehrsmanagement anhand positionsbasierter

[47] *Lieferung mit Drohnen, Lastenrädern oder Elektrofahrzeugen, Sharing-Optionen sowie die Nutzung öffentlicher Verkehrsmittel außerhalb der Stoßzeiten.*

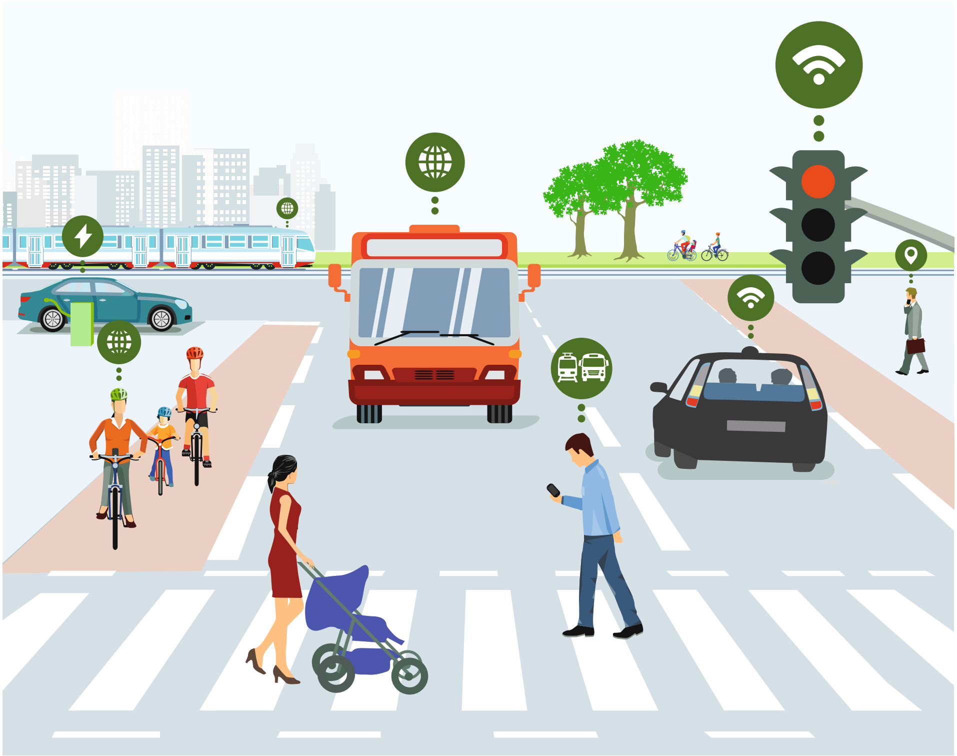

*Abb. 7.2 Digitalisierung, Vernetzung und Automatisierung sind die Schlüsseltechnologien für die Gestaltung einer nachhaltigen Mobilität. (Quelle: scusi/AdobeStock)*

Informationen sowie der Einsatz von KI-Methoden zur adaptiven Steuerung und der intelligenten Energieerzeugung und -speicherung. Ein effizientes Gesamtsystem erfordert in der Regel ein Mindestmaß an Vernetzung zwischen den beteiligten Akteuren sowie gegebenenfalls mit der Infrastruktur und ermöglicht zudem die Nutzung der Vorteile von Automatisierungstechnologien.

Neben der Förderung entsprechender technologischer als auch physischer Infrastruktur[48] zur Umsetzung einer verlässlichen und nahtlosen „Tür-zu-Tür"-Mobilität[49] sind entschlossene politische Vorgaben und ein konsequentes Umdenken des bisherigen

[48] *Sensorik, Ladeinfrastruktur, Umsteigepunkte, multimodale Plattformen etc.*

[49] *Inklusive effizienter Routenplanung, transparenter Ticketkauf, Echtzeit-Reiseinformation, personalisierte On-Demand-Angebote etc.*

Emissionsverhaltens[50] notwendig. Weiterhin ist ein Paradigmenwechsel von „schnell und allein genutzt“ hin zu „emissionsfrei, effizient, sicher, adaptiv, nahtlos, zugängig, ökonomisch nachhaltig, flexibel und verlässlich“ Voraussetzung für die Umsetzung einer nachhaltigen Mobilität. Dieser Ansatz verabschiedet sich vom Streben nach individuellem Fahrzeugbesitz, betont kollektive Funktionen und zeichnet sich zudem durch eine multimodale und interoperable Durchlässigkeit zwischen den Verkehrsträgern aus und zwar sowohl im Personen- wie im Güterverkehr.

Ökonomische Steuerungsmechanismen wie dynamische Straßennutzungsgebühren, Parkraummanagement und höhere Steuern für Autobesitzer sowie Fahrverbote in Innenstädten und Tempo 30 als Regelgeschwindigkeit innerorts könnten eine wichtige Rolle bei der Förderung von „lebenswerten Städten“ spielen (Randelhoff 2019; Agora Verkehrswende 2018) und somit zur besseren räumlichen und zeitlichen Verteilung der Verkehrsströme sowie einer Verlagerung auf andere Verkehrsmittel beitragen.

Der für die Preisgestaltung notwendige Vergleich der gesamtgesellschaftlichen Kosten kann in vielen Punkten direkt erfolgen, zum Beispiel beim Vergleich des urbanen Platzbedarfs eines Verkehrsmittels pro Nutzer:in. Aber auch indirekte Indikatoren, wie etwa die Bewertung der Gesundheitskosten, können zum Ansatz gebracht werden, denn ein nachhaltiges Mobilitätssystem sollte die ökonomischen, sozialen und ökologischen Anforderungen verschiedener Nutzungsgruppen berücksichtigen und im ökologisch vertretbaren Rahmen bestmöglich bedienen. Die Gestaltung des Mobilitätssystems sollte daher im Dialog zwischen allen beteiligten Gruppen und mit dem Ziel der Erreichung der Nachhaltigkeitsziele erfolgen. Und da die Lage eine abrupte Verkehrswende erfordert, sollte die Diskussion nicht am Status quo ausgerichtet sein und allen Gruppen eine gleichberechtigte Teilhabe ermöglichen. Kommunale Entscheidungsträger:innen müssen bereit sein, die notwendigen Schritte zur Stärkung nachhaltiger Mobilitätsformen einzuleiten.

Dass allerdings der Wandel eines über Jahrzehnte gewachsenen Verkehrssystems nicht ohne Weiteres über Nacht gelingt, zeigen die vielen Maßnahmen, die kurzfristig bei der Lockerung des gesellschaftlichen Stillstands während der Covid-19-Pandemie eingeleitet wurden (POLIS 2020; Laker 2020). Die Handlungsfähigkeit staatlicher Institutionen kann erhöht werden, wenn die freie Mobilitätsdatenverfügbarkeit sichergesellt ist. Als Vorbild kann zum Bespiel der vom Los Angeles Department of Transportation (LADOT) initiierte und von der kommunengestützten „Open

[50] *Bei der Stromerzeugung wurde beispielweise zunächst auf den (staatlichen) Ausbau erneuerbarer Energiequellen und, sobald ein Niedrigemissionsangebot geschaffen wurde, auf überwiegend nach wirtschaftlichen Gesichtspunkten getroffenen (Grundsatz-)Entscheidungen zur Energiewahl auf Nutzerseite gesetzt.*

Mobility Foundation“ entwickelte Standard „Mobility Data Specification“ (MDS) (Open Mobility Foundation 2018) für den Datenaustausch zwischen Stadtverwaltungen und Mobilitätsanbietern dienen. Erste Ansätze zur Nutzung sind auch bereits in Europa (speziell Deutschland) zu finden (Radforschung 2019). Freie Datenverfügbarkeit, aber auch freie (Open-Source-)Lizenzen für Software und Quellcode können einerseits durch die breite Beteiligung der Öffentlichkeit die Entwicklung neuer und nachhaltiger Mobilitätsangebote beschleunigen und andererseits das Risiko eines (privaten) Angebotsmonopols minimieren (Behrendt und Bormann 2020). Dieses Vorgehen kann politisch bestärkt werden, wenn die Förderung im Mobilitätsbereich an die freie Daten- und Quellcodebereitstellung gekoppelt wird (Kirschner 2020). In Europa strebt beispielsweise die „MaaS Alliance“[51] die Integration verschiedener Verkehrsmittel in ein übergeordnetes On-Demand-Mobilitätssystem unter Einbezug von Verkehrsunternehmen, Dienstleistern, Kommunen und Nutzer:innen an.

Aber nicht nur innerhalb des Verkehrssektors gilt es, Synergiepotenziale aufzuzeigen und zu nutzen. Sektorenkopplung ist ein wichtiger Faktor für die Senkung der Treibhausgasemissionen durch Substitution fossiler Energieträger mit (primär) erneuerbaren in den Sektoren Wärme, Mobilität und Industrie. Sekundäre Ziele der Sektorenkopplung können in der Nutzung von Freiheitsgraden der Optimierung innerhalb eines zunehmend und perspektivisch vollständig dekarbonisierten Energie- und Wirtschaftssystems sowie durch einen Beitrag zur Flexibilisierung und Energieeffizienzsteigerung entstehen (Winter 2018). Im Verkehrssektor gibt es die folgenden Sektorenkopplungsoptionen: direkte Nutzung von erneuerbarem Strom – mit und ohne Zwischenspeicherung, indirekte Nutzung von erneuerbarem Strom zur Erzeugung von Kraftstoffen (zum Beispiel PtL, PtG) und Wasserstoff ($PtH_2$) und Biomasse (Wietschel 2019). Zudem tragen Sektorenkopplungsoptionen zur Erhöhung der Energieeffizienz und durch ihr hohes Flexibilitätspotenzial zur Systemintegration von erneuerbaren Energien bei.

Nach einem halben Jahrhundert, in dem Mobilität von Globalisierung und Wachstum bestimmt und nicht (ausreichend) nachhaltig ausgerichtet wurde, bewirkt sie deutliche Klimaschäden, ist unzureichend inklusiv und verringert besonders in konzentrierten urbanen Räumen die Lebensqualität. Emissionsarme Antriebstechnologien sowie Digitalisierung und Automatisierung können, klug genutzt, zu einer nachhaltigen Mobilität beitragen.

[51] *https://maas-alliance.eu/.*
